# Host targets of candidalysin

**DOI:** 10.1371/journal.ppat.1013284

**Published:** 2025-06-23

**Authors:** Jianfeng Lin, Scott G. Filler

**Affiliations:** 1 Division of Infectious Diseases, Lundquist Institute for Biomedical Innovation at Harbor-UCLA Medical Center, Torrance, California, United States of America; 2 David Geffen School of Medicine at University of California Los Angeles, Los Angels, California, United States of America; University of Maryland, Baltimore, UNITED STATES OF AMERICA

## Abstract

*Candida albicans* is a normal constituent of the human microbiota and a ubiquitous human threat. This fungus causes diseases ranging from superficial cutaneous and mucosal candidiasis to life-threatening disseminated candidiasis. *C. albicans* hyphae secrete candidalysin, a cytolytic peptide toxin that damages host cells and activates immune responses. Candidalysin plays a key role in both pathogenicity and commensalism. In this Pearl, we review the host targets of candidalysin and how they modulate the interaction of *C. albicans* with the host.

## 1. Introduction

In 2016, the Naglik and Hube groups reported that *Candida albicans* hyphae secrete candidalysin, a 31-amino acid cytolytic peptide toxin that is released from the Ece1 protein by the action of the Kex1/Kex2 proteases [1]. This toxin has been found to play critical roles in *C. albicans* virulence during oropharyngeal, vulvovaginal, and hematogenously disseminated candidiasis, as well as colonization of the gastrointestinal tract [1–4]. Central to the function of candidalysin is its ability to insert into the plasma membrane and form pores that disrupt osmotic balance and trigger the lysis of red blood cells, epithelial cells, and macrophages [1,5,6]. Candidalysin likely forms multimers that organize into a pore that subsequently inserts into the membrane with the integral facilitation of the non-candidalysin-Ece1-peptides (NCEPs) [7,8]. It has been found that candidalysin can form pores in artificial lipid bilayers composed of dioleoylphosphatidylcholine (DOPC) [1,7]. These results indicate that candidalysin can form membrane pores in the absence of a cell surface receptor.

**Fig 1:**
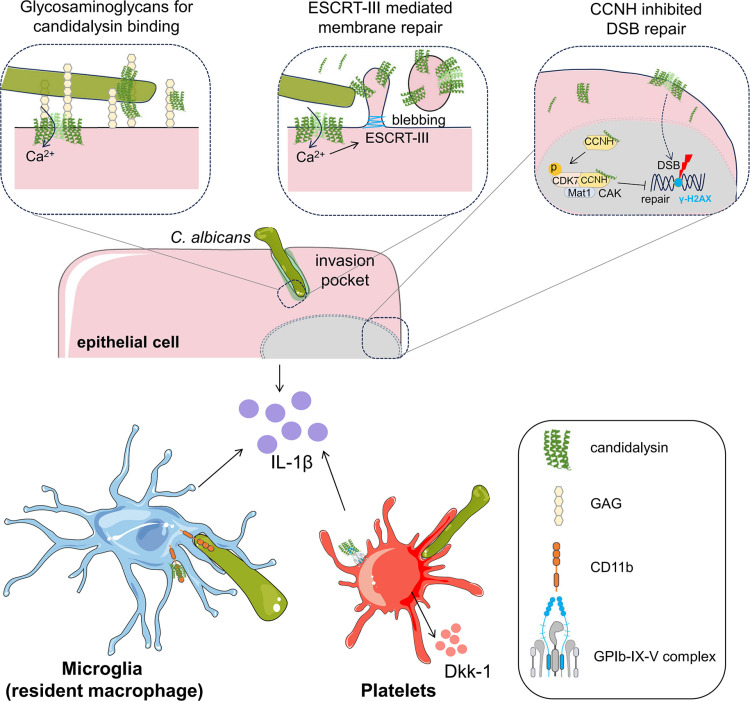
Host cell targets of candidalysin. During the invasion of epithelial cells by *Candida albicans*, an invasion pocket is formed around the invading hypha, in which the secreted candidalysin pore-forming toxin accumulates. Candidalysin binds to glycosaminoglycans (GAGs) on the host cell surface, which promote its polymerization and facilitate its insertion into the host cell membrane. The inserted candidalysin pores cause an influx of calcium ions that activates the ESCRT-III pathway that induces membrane repair by blebbing. Inside the cell, candidalysin induces DNA double-strand breaks (DSBs) through mechanisms that remain unclear. It binds to cyclin H (CCNH), activates the cyclin-dependent kinase (CDK)-activating kinase (CAK) complex by phosphorylating CDK7, which subsequently inhibits the repair of DSBs. Candidalysin stimulates epithelial cells to secrete proinflammatory cytokines and chemokines that recruit macrophages and neutrophils to the infection site to combat the fungus. In brain microglial cells, candidalysin interacts with CD11b on these cells to amplify the immune response and enhance fungal clearance from the brain. During *C. albicans* colonization of the airways, candidalysin interacts with GPIbα on platelets, stimulating the release of Dickkopf-1 (Dkk-1) which induces Th2 and Th17 responses, leading to reactive airway disease. The artwork used in the main figure was adapted from Servier Medical Art (https://smart.servier.com/). Servier Medical Art is licensed under a Creative Commons Attribution 4.0 International License (CC BY 4.0).

Emerging research challenges the assumption of that pore-forming toxins such as candidalysin function independently of host cell proteins or glycans. For instance, bacterial pore-forming toxins such as cholesterol-dependent cytolysins or aerolysin-like toxins were long thought to act indiscriminately, yet receptors such as glycoproteins or glycans are now recognized as key facilitators of toxin activity [9]. Here, we review recent studies demonstrating that candidalysin directly interacts with host surface molecules and intracellular pathways to amplify its effects (Fig 1).

### 2. Epithelial cell targets of candidalysin

#### 2.1. Glycosaminoglycans are the primary surface-expressed targets.

Results of recent studies indicate that host receptors play a key role in candidalysin function. We performed a genome-wide CRISPR/Cas9 loss-of-function screen in oral epithelial cells and found that disruption of glycosaminoglycan (GAG) biosynthesis conferred significant resistance to candidalysin-induced cytotoxicity [10]. GAGs are linear polysaccharides composed of repeating disaccharides groups that are often sulfated. Cell surface GAGs are covalently bound to core proteins via a tetrasaccharide linker. This linker is synthesized by the sequential activity of four enzymes. The CRISPR/Cas9 screen indicated that disruption of genes encoding three of these enzymes, *XYLT2*, *B3GALT6*, or *B3GAT3* resulted in the complete absence of GAGs on the epithelial cell surface and reduced susceptibility to damage caused by both candidalysin and live *C. albicans.* These results demonstrate the critical role of cell surface GAGs in facilitating the cell-damaging effects of candidalysin [10].

Although cells lacking surface GAGs were more resistant to candidalysin-induced damage, this resistance could be overcome by higher concentrations of candidalysin [10]. This result aligns with biophysical evidence showing that candidalysin can form pores in artificial DOPC membranes that lack GAGs [1,7].

Immunofluorescence microscopy demonstrated that candidalysin colocalized with sulfated GAGs on epithelial cells. This interaction was confirmed by surface plasmon resonance (SPR), which indicated that candidalysin bound to heparin (a ubiquitous sulfated GAG) with nanomolar affinity [10]. When GAG-deficient cells were treated with candidalysin, calcium influx—a hallmark of pore formation—was markedly blunted, suggesting that GAGs likely act as scaffolds for toxin oligomerization, membrane insertion, and/or pore stabilization. It was also found that treating cells with exogenous GAGs protected them from damage. One particularly effective GAG analog was dextran sulfate, which provided strong protection against candidalysin-induced damage to epithelial cells in vitro. In the mouse model of vulvovaginal candidiasis, dextran sulfate reduced vaginal cell damage and decreased inflammation but had no effect on vaginal fungal burden. The effects of dextran sulfate phenocopied vaginal infection with the candidalysin-deficient *ece1*Δ/Δ deletion mutant. These findings highlight GAGs as critical host cofactors that enhance candidalysin activity in vivo and in vitro.

Epithelial cells express multiple GAGs, including heparan sulfate, chondroitin sulfate, and dermatan sulfate. Deletion of genes whose products are responsible for synthesizing the tetrasaccharide linker results in the absence of all GAGs on the cell surface. It is currently unknown which GAGs are the main targets of candidalysin and whether specific cell surface proteins linked to these GAGs mediate the effects of candidalysin on epithelial cells and/or other host cells.

#### 2.2. The ESCRT III pathway repairs candidalysin-induced membrane damage.

Westman and colleagues [11] discovered that epithelial cells actively resist candidalysin-induced damage by forming membrane blebs. They determined the calcium influx induced by candidalysin membrane pores triggers the hydrolysis of phosphatidyl inositol-4,5-biophosphate (PtdIns(4,5)P₂), which causes breakdown of cortical actin and destabilizes the cell membrane. The calcium influx also activates the Alg-2/Alix/ endosomal complexes required for transport (ESCRT)-III pathway, which induces the formation of membrane blebs. This process enables cells to shed compromised membrane sections containing candidalysin, preventing further toxicity. Consistent with this mechanism, Lin et al. found that candidalysin-treated epithelial cells progressively lost cell surface GAGs [10], which were presumably shed via membrane blebbing.

The epithelial cell plasma membrane is also damaged when epithelial cells are invaded by progressively elongating hyphae. This type of damage occurs independently of candidalysin. Westman and colleagues [11] found evidence that these plasmalemmal tears also trigger calcium release, leading to lysosome exocytosis. Through this process, lysosomal membranes can fuse with the plasma membrane, potentially sealing mechanical breaches and restoring epithelial integrity. Also, host-derived extracellular vesicles can sequester candidalysin and protect cells against the toxin [8]. It is possible that the released candidalysin-rich membrane vesicles may function as danger/alarm signals for nearby cells and thus amplify immune responses. However, this hypothesis has not been tested.

#### 2.3. Cyclin H (CCNH) mediates intracellular DNA damage.

To identify intracellular targets of candidalysin, Zhang and colleagues performed high-throughput enhanced yeast two-hybrid screening [12]. The results of this screen identified cyclin H (CCNH) as a critical intracellular target of candidalysin. CCNH is a regulatory subunit of the cyclin-dependent kinase (CDK)-activating kinase (CAK) complex, which is essential for DNA damage repair. The binding candidalysin to CCNH was confirmed by co-immunoprecipitation, bimolecular fluorescence complementation, and SPR. This binding activates the CAK complex to phosphorylate CDK1/2, which inhibits DNA damage repair. It was found that intracellular candidalysin not only induces DNA double-strand breaks but suppresses host DNA repair pathways. Through these mechanisms, candidalysin exacerbates genomic instability, as evidenced by increased epithelial cell expression of phosphorylated histone H2AX, a marker of DNA double strand breaks. These in vitro results were verified in a mouse model of oropharyngeal candidiasis, where infection with wild-type *C. albicans* caused DNA double strand breaks in epithelial cells, while a candidalysin-deficient *ece1*Δ/Δ mutant failed to induce detectable DNA damage.

It has been proposed that oral carriage of *C. albicans* is a risk factor for oral squamous cell carcinoma (reviewed in [13]). One potential mechanism for this association is that *C. albicans* can stimulate the epidermal growth factor receptor and c-met [14], oncogenic receptors that are frequently activated in epithelial cell cancers. The results of Zhang and colleagues suggest the intriguing possibility that *C. albicans* could also enhance oncogenesis by causing DNA damage and inhibiting its repair [12].

### 3. Integrin CD11b is a key target for candidalysin on microglial cells

Patients with deficiency in the C-type lectin receptor-Syk (spleen tyrosine kinase) adaptor CARD9 are at increased risk of fungal infection in the brain. Drummond and colleagues found that during *C. albicans* brain infection, CARD9-expresssing microglial cells produce IL-1β, which stimulates these cells to make CXCL1 [15]. This chemokine recruits monocytes and neutrophils to the brain, leading to clearance of the infecting fungus. Candidalysin is the key *C. albicans* factor that drives IL-1β release, as an *ece1*Δ/Δ mutant fails to induce production of IL-1β and CXCL1 [6], resulting in reduced recruitment of monocytes and neutrophils, and elevate brain fungal burden. Recently, Wu and colleagues discovered that CD11b (integrin αM) functions as the microglial target that binds to and is activated by candidalysin [16]. They found that candidalysin directly binds to CD11b. This binding stimulates microglia to inhibit *C. albicans* germination and secrete pro-inflammatory cytokines. This interaction is essential for eliminating fungal cells from the brain, as mice with CD11b-deficient microglia have reduced *C. albicans* clearance from the brain. These results indicate that the interaction of candidalysin with CD11b on microglial cells plays a central role in the host defense against *C. albicans* infection of the brain. It is known that CD11b is expressed by other phagocytes, such as neutrophils, monocytes, and macrophages, and that candidalysin can activate these cells [4,17–19]. However, it is not yet known whether candidalysin activates these other phagocytes by binding to CD11b.

### 4. Platelet GPIbα binds candidalysin and drives allergic airway disease

A subset of asthma patients has reactive airway disease due to airway colonization with *C. albicans.* Wu and colleagues studied this phenomenon in a mouse model of *C. albicans* airway sensitization [20]. They found that candidalysin is the key factor secreted by the fungus that stimulates Th2 and Th17 immune responses in the lung. Candidalysin directly binds to GPIbα, a subunit of the platelet receptor complex GPIb-IX-V. This binding induces platelet activation, aggregation, and the release of pro-inflammatory mediators including Dickkopf-1 (Dkk-1). Dkk-1 promotes Th2 and Th17 immune polarization, driving allergic inflammation in the mouse fungal asthma model. Platelet-derived cytokines such as IL-1β recruit and activate neutrophils and monocytes, worsening immunopathology. Blocking GPIbα or downstream Dkk-1 signaling reduces Th2/Th17 responses in the asthmatic mice. These results suggest that strategies to mitigate candidalysin-driven inflammation may be efficacious in asthma patients who are colonized with *C. albicans* [20].

## 5. Conclusions and future directions

To date, only a limited number host cell targets of candidalysin have been identified, epithelial cell GAGs and cyclin H, microglial cell CD11b, and platelet GPIbα. The diversity of these targets suggests that candidalysin may interact with additional host molecules yet to be identified. While it is known that GAGs are required for candidalysin to both stimulate and damage epithelial cells, the function of CD11b and GPIbα has only been studied in the context of immune stimulation. Thus, it remains to be determined whether deletion of CD11b from phagocytes or GPIbα from megakaryocytes and platelets will protect these cells from candidalysin-induced damage. From a therapeutic standpoint, it is clear that blocking the interaction of candidalysin with its host cell targets can have vastly different effects, depending on the site of infection. Inhibiting this interaction is beneficial in mucosal candidiasis and *C. albicans*-induced reactive airway disease but detrimental during *C. albicans* infection of the brain. Thus, the duality of candidalysin activity needs to be accounted for when considering therapeutic strategies directed against this pore-forming toxin.
